# First person – Jeanette C. Perron

**DOI:** 10.1242/bio.045781

**Published:** 2019-07-15

## Abstract

First Person is a series of interviews with the first authors of a selection of papers published in Biology Open, helping early-career researchers promote themselves alongside their papers. Jeanette C. Perron is first author on ‘Chemotropic signaling by BMP7 requires selective interaction at a key residue in ActRIIA’, published in BiO. Jeanette is an assistant professor at St John's University, USA, investigating the specificity of BMP-dependent intracellular signaling in neural development and disease.


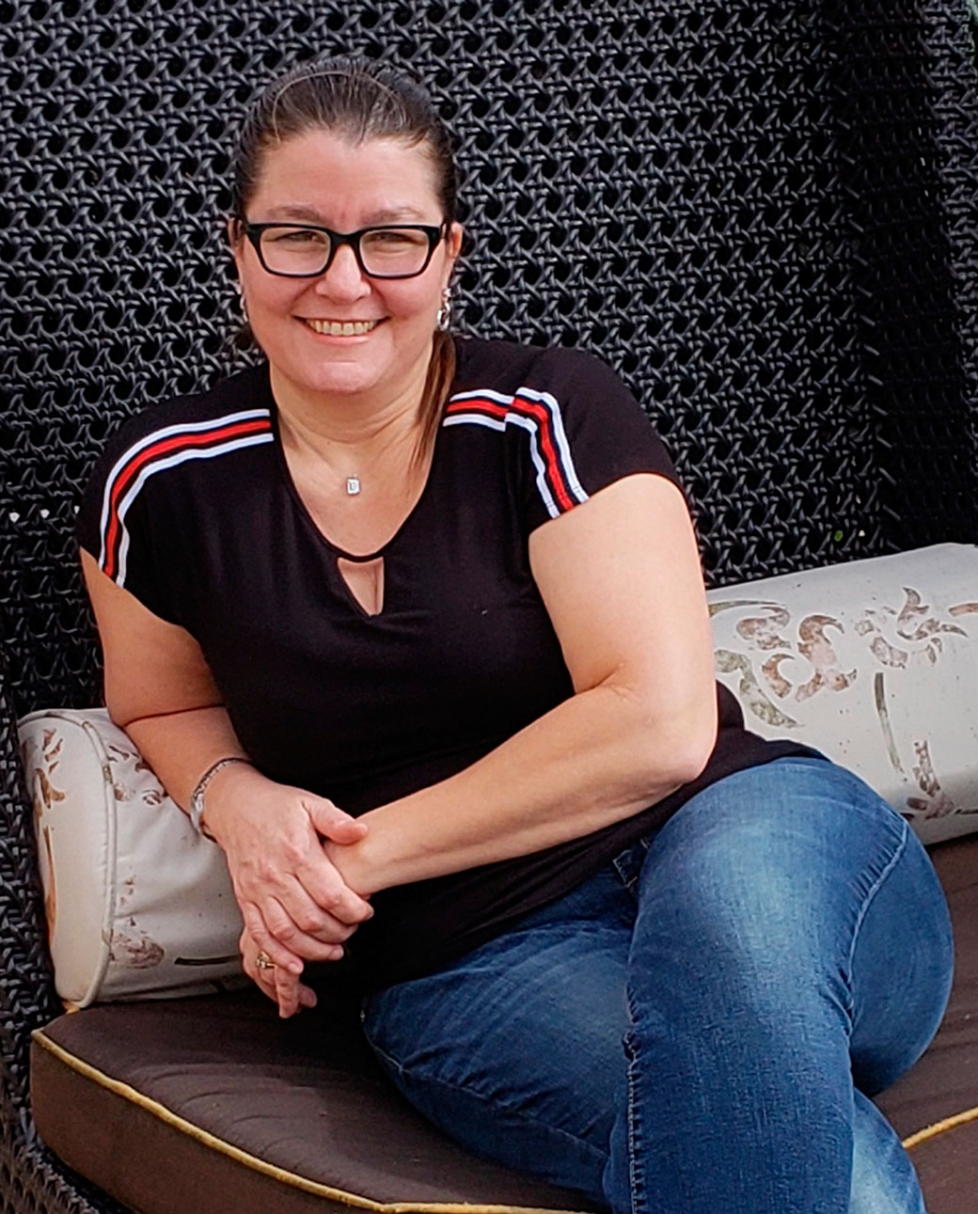


**Jeanette C. Perron**

**What is your scientific background and the general focus of your lab?**

My background in science has focused primarily on developmental neurobiological problems and the intracellular signaling events that regulate axon growth and guidance. I earned a Ph.D. from the University of Miami School of Medicine, completed postdoctoral training and worked as an associate research scientist at Columbia University and now have recently started my own lab at St John's University. My lab is interested in dissecting the intracellular signaling mechanisms regulated by the large family of cellular factors known as bone morphogenetic proteins (BMPs). These factors are critical for early embryonic development, differentiation and patterning of neural progenitor cells, axon orientation and guidance, and even synapse formation. BMPs control all of these important actions and many more through a small subset of receptor subunits arranged in a tetrameric receptor complex. Moreover, diseases as diverse as hereditary brachydactyly and familial pulmonary hypertension are caused by mutations in BMP receptor subtypes. How different BMPs recruit the various receptor subunits to direct these different activities is a challenging problem and understanding the specificity underlying these complex signaling pathways is essential for the development of potential therapeutic agents that would allow us to control the action of these multifaceted factors.

**How would you explain the main findings of your paper to non-scientific family and friends?**

Some proteins made in the body are able to change how cells behave or even dictate what type of cell that cell is fated to become. BMPs are a family of such proteins. BMPs interact with a small subset of proteins or BMP receptors found on the surface of cells. The interactions between BMPs and their receptors result in the activation of many different signals that travel through the cell by different pathways effecting cellular behavior. In our study, we found that a specific set of BMP receptor subunits is required for producing signals necessary for cellular movement, such as attracting cells of the immune system to sites of injury or guiding axons to their targets during nervous system development. Loss of function of these receptor subunits significantly affected only one of the pathways stimulated by BMPs, allowing us to separate and potentially control the different messages emanating from BMP receptors. Remarkably, we also found that one small change in the structure of one of the BMP receptors can eliminate one type of response while leaving other responses intact. These data indicate that precise structural characteristics of BMPs and their interactions with BMP receptors can be manipulated to achieve the desired response.

“Some proteins made in the body are able to change how cells behave or even dictate what type of cell that cell is fated to become.”

**What are the potential implications of these results for your field of research?**

Our data are bringing us closer to understanding how to control the complex signaling mechanisms utilized by BMPs. This information can lead to the development of therapeutic approaches to address diseases or disorders in which BMPs, their receptors or BMP-dependent signaling mechanisms are altered.

**What has surprised you the most while conducting your research?**

It is amazing to consider how changing a single amino acid in one of the type II BMP receptors, ActRIIA, could drastically change the functional response to BMP7. BMP6 is a BMP that cannot activate the full array of signaling downstream of BMP receptor activation. In previous work, we showed that a single change in the amino acid sequence encoding BMP6 imparts cellular responses not previously achieved by BMP6 stimulation. These findings imply that highly specific agonists could be developed to precisely harness or inhibit the activity of distinct signaling pathways downstream of BMP receptors, while allowing signaling through other pathways to take place unimpeded.

**Figure:**
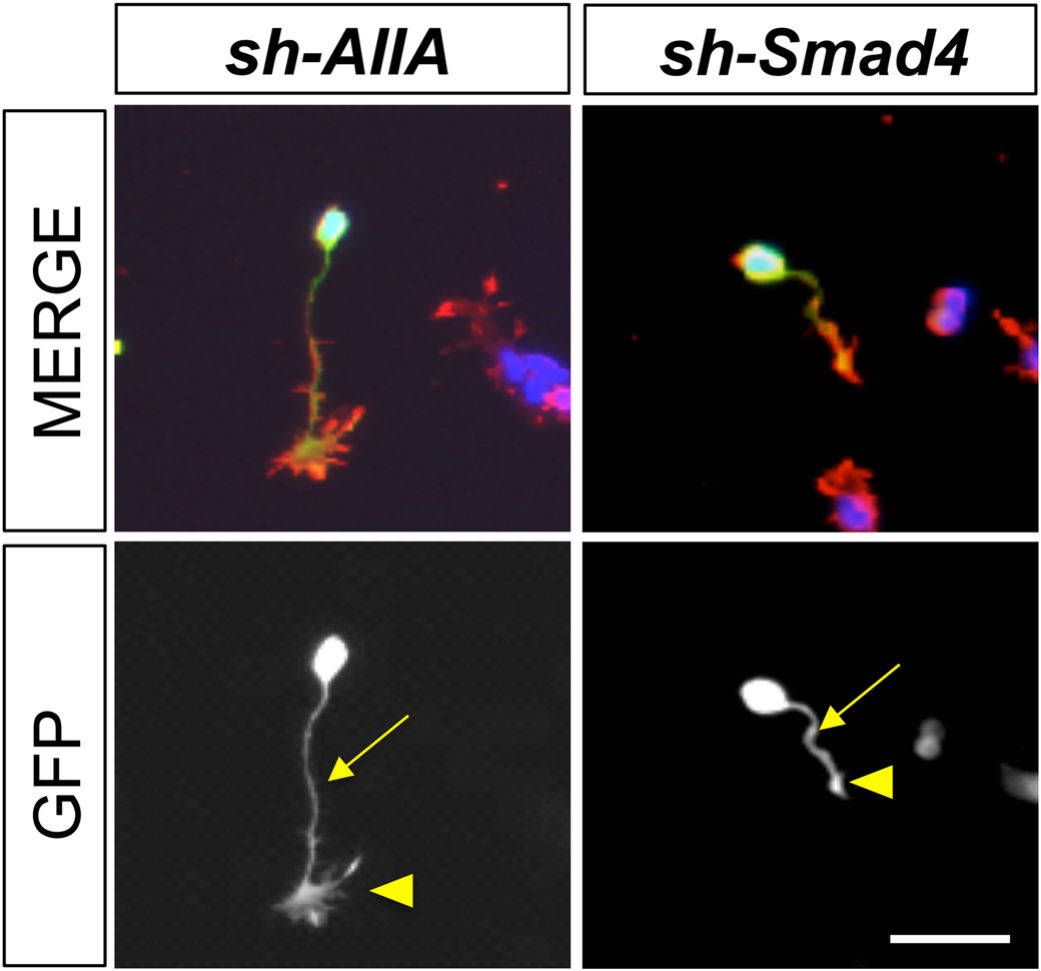
**Knockdown of type II BMP receptor, ActRIIA, with *sh-AIIA* prevents growth cone collapse by BMP7, whereas inhibiting type I BMP receptor-dependent signaling with *sh-Smad4* has no effect on BMP7-evoked growth cone collapse.**

**What, in your opinion, are some of the greatest achievements in your field and how has this influenced your research?**

I have long been fascinated by the complexity of intracellular signaling pathways. The appreciation of how protein phosphorylation and protein–protein interactions control cellular responses to extracellular factors has provided an explosion of information and has directed researchers to look for signaling specificity at ligand/receptor interactions, as well as, importantly, downstream of these interactions. The fact that many of these factors play multiple roles in embryonic development, which are often different from the actions these factors perform in the adult organism, inspires the search for signaling specificity that underlies my research.

“The appreciation of how protein phosphorylation and protein–protein interactions control cellular responses to extracellular factors has provided an explosion of information…”

**What's next for you?**

Following the publication of this paper, I am focused on building on the successes of my new laboratory. I look forward to training more excellent graduate students, publishing solid, meaningful papers, writing grants to fund our research and attending meetings to promote our work. As an assistant professor on the tenure track, what's next is making sure that I work hard so I can continue having the best job in the world!
